# Safety Assessment of *Lactiplantibacillus plantarum* GUANKE Based on Whole-Genome Sequencing, Phenotypic, and Anti-Inflammatory Capacity Analysis

**DOI:** 10.3390/microorganisms13040873

**Published:** 2025-04-10

**Authors:** Simin Lu, Kun Yue, Siqin He, Yuanming Huang, Zhihong Ren, Jianguo Xu

**Affiliations:** National Key Laboratory of Intelligent Tracking and Forecasting for Infectious Diseases, National Institute for Communicable Disease Control and Prevention, Chinese Center for Disease Control and Prevention, Beijing 102200, China

**Keywords:** *L. plantarum*, whole-genome sequencing, immunological regulation

## Abstract

*Lactiplantibacillus plantarum* GUANKE (*L. plantarum* GUANKE) is a Gram-positive bacterium isolated from the feces of healthy volunteers. Whole-genome sequencing analysis (WGS) revealed that the genome of *L. plantarum* GUANKE consists of one chromosome and two plasmids, with the chromosome harbors 2955 CDS, 66 tRNAs, and 5 rRNAs. The genome is devoid of virulence factors and Clustered regularly interspaced short palindromic repeats (CRISPR)/CRISPR-associated (Cas) systems. It contains three intact prophage regions and bacteriocin biosynthesis genes (plantaricins K, F, and E), as well as seventeen genomic islands lacking antibiotic resistance or pathogenicity determinants. Functional prediction outcomes identified that the genome of *L. plantarum* GUANKE is closely related to transcription, carbohydrate transport and metabolism, and amino acid transport and metabolism. Carbohydrate-active enzymes (CAZymes) analysis and GutSMASH analysis revealed that the genome of *L. plantarum* GUANKE contained 100 carbohydrate-active enzyme genes and two specialized metabolic gene clusters. Safety assessments confirmed that *L. plantarum* GUANKE neither exhibited β-hemolytic activity nor harbored detectable transferable drug resistance genes. The strain exhibited remarkable acid tolerance and bile salt resistance. Cellular adhesion assays demonstrated moderate binding capacity to Caco-2 intestinal epithelium (4.3 ± 0.007)%. In vitro analyses using lipopolysaccharide (LPS)-stimulated macrophage models demonstrated that *L. plantarum* GUANKE significantly suppressed the secretion of pro-inflammatory cytokines (TNF-α, IL-6, IL-1β), exhibiting dose-dependent anti-inflammatory activity. In vivo experiments showed that *L. plantarum* GUANKE was involved in the regulation of the apical junction pathway and interferon pathway in colon tissue of normal mice.

## 1. Introduction

Probiotics are a kind of live microorganism that, when administered in adequate amounts, confer a health benefit on the host. It has been proposed that the functions of probiotics at the genus level are to inhibit the intestinal colonization of pathogenic bacteria, restore the unbalanced intestinal microbiota, produce short-chain fatty acids or other acidic substances, and increase the turnover of intestinal cells. Synthesizing vitamins, metabolizing bile salts, strengthening the intestinal barrier, and neutralizing carcinogens are the functions of probiotics at the species level. Regulation of the nervous system, the immune system and the endocrine system, and the production of specific bioactive substances are the functions of probiotics at the strain level [1].

With the widespread use of probiotics in healthy people and clinical patients, the safety of probiotics has attracted increasing attention. Because probiotics can survive in the host, probiotics are thought to have the potential to be infectious and/or produce toxic substances. According to a report jointly published by the Food and Agriculture Organization of the United Nations (FAO) and the World Health Organization (WHO), probiotics may produce four side effects: systemic infection, harmful metabolic activity, excessive immune stimulation of susceptible individuals, and gene transfer [2]. For example, available clinical case reports suggest that bacteremia may be triggered by the use of *Lacticaseibacillus rhamnosus* GG intervention in immunosuppressed patients with severely active ulcerative colitis [3]. Therefore, as an emerging microbial therapy, safety evaluation of potential probiotic candidate strains is a crucial prerequisite for the application of probiotics.

WGS is a high-throughput sequencing technology that enables the systematic analysis of the complete genome of microorganisms or host organisms. As a key detection technology, WGS can become the core link in probiotic safety assessments by accurately identifying the genetic characteristics of probiotic strains and systematically screening virulence factor genes, disease-related genes, and antibiotic resistance genes [4]. For example, WGS analysis revealed the presence of prophage regions and CRISPR-Cas regions in the genome of *L. plantarum* cqf-43, as well as the absence of known virulence genes, toxin genes, hemolysin genes, gelatinase genes, bioamine production genes, and detectable transferable resistance genes [5]. The genome of *Lactococcus lactis* subsp. *lactis* LL16 encompasses type-III polyketone synthase (T3PKS), which generates the putative bacteriocins lactococcin B and enterolysin A, as well as genes encoding the neurotransmitters serotonin and gamma-aminobutyric acid [6]. KEGG (Kyoto Encyclopedia of Genes and Genomes) pathway and CAZymes analysis of the genome of *L. pentose* L33 showed that *L. pentose* L33 has seven amino acids biosynthesized pathways, while it can degrade a variety of carbohydrates [7]. In addition, evaluation of the cytotoxicity of probiotics to intestinal cells, hemolysis analysis, and antibiotic sensitivity analysis of probiotics are also important means to evaluate the safety of probiotics. Besides demonstrating safety performance, assessing the ability of microbes to withstand acidic conditions and bile salts, as well as their ability to adhere to cells, is also an important means of screening potential probiotics.

*L. plantarum* is a facultative anaerobic bacterium that is widely distributed in the human gastrointestinal tract and fermented foods, belonging to the *Lactiplantibacillus* genus within the *Lactobacillaceae* Firmicutes family. Research has demonstrated that *L. plantarum* possesses various functional characteristics, and its functionality is strain-specific, encompassing antagonistic activity against pathogenic microorganisms, restoration of microbiota dysbiosis, participation in host metabolism, and modulation of immune responses [8,9,10,11,12,13,14]. For example, *L. plantarum* L168 and its derived metabolite indole-3-lactic acid enhance the antitumor immunosuppression of CD8^+^ T cells through epigenetic regulation [12]. The *L. plantarum* KABP011, KABP012, and KABP013 effectively modulate bile acid and cholesterol metabolism in overweight individuals, thereby reducing ApoB and small Low-density lipoprotein levels, decreasing Low-density lipoprotein sensitivity to oxidation, and enhancing High-density lipoprotein antioxidant capacity [13]. After intervention in malnourished infants, *L. plantarum* Dad-13 demonstrated a significant ability to promote the growth of butyric acid-producing bacteria while simultaneously inhibiting the growth of Enterobacteriaceae bacteria in the infants’ gut [14]. In this study, to evaluate the suitability of *L. plantarum* GUANKE, a probiotic isolated from the feces of healthy humans, we analyze its genomics, prebiotic characteristics, anti-inflammatory ability, and immune modulation ability.

## 2. Materials and Methods

### 2.1. Bioinformatic Analysis of the Genome of L. plantarum GUANKE

The complete genome sequence of *L. plantarum* GUANKE (NCBI Species ID: 1300221; Accession: GCA_000604105.1) was downloaded from the NCBI database. A ring map of the genome of *L. plantarum* GUANKE was generated using Proksee online software (https://proksee.ca/, accessed on 15 December 2024). The Virulence factor database (VFDB) [15] and Comprehensive Antibiotic Resistance Database (CARD) [16] were used to predict the possible virulence genes and drug resistance genes in the *L. plantarum* GUANKE genome online with default parameters. CRISPR-Cas systems, prophage regions, bacteriocin clusters, and genomic islands were identified using CRISPRCasFinder [17], Phage Search Tool-Enhanced Release (PHASTER) [18], BAGEL4 [19], and IslandViewer [20], respectively, with standard analysis settings.

### 2.2. Functional Genome Annotation

Functional annotation of the *L. plantarum* GUANKE genome was conducted using eggNOG-mapper v2.1.3 with the eggNOG 5.0 database [21]. Enzymes associated with carbohydrate utilization in the *L. plantarum* GUANKE genome were analyzed online using the CAZymes (carbohydrate-active enzymes) database [22]. GutSMASH was used to identify, annotate, and analyze specific gene clusters associated with primary metabolism in the *L. plantarum* GUANKE genome using the default parameters [23].

### 2.3. Bacteria and Cell Culture

As previously described [24], *L. plantarum* GUANKE was cultured in MRS (De Man–Rogosa–Sharpe, Thermo Scientific™ Oxoid, Basingstoke, UK) medium. Human intestinal epithelial cell line Caco-2 was cultured in Dulbecco’s Modified Eagle Medium (DMEM, Gibco, Grand Island, NY, USA) containing 100 U/mL penicillin (Gibco, Grand Island, NY, USA), 100 U/mL streptomycin (Gibco, Grand Island, NY, USA), and 10% fetal bovine serum (FBS, Thermo Fisher, Waltham, MA, USA). Human mononuclear leukemia cell line (THP-1) was cultured at 37 °C, 5% CO_2_ in RPMI-1640 medium (Gibco, Grand Island, NY, USA) supplemented with 100 U/mL penicillin, 100 U/mL streptomycin, 0.05 mM 2-mercaptoethanol (Sigma, St. Louis, MO, USA), and 10% FBS. THP-1 cells (5 × 10^5^) were seeded in 24-well cell culture plates, pretreated with 100 ng/mL PMA (phorbol 12-myristate 13-acetate, Sigma, St. Louis, MO, USA) for 24 h to induce adherent macrophages, washed in PBS twice, and placed in fresh medium without penicillin-streptomycin and PMA for 6 h for follow-up experiments.

### 2.4. Phenotypic Characterization

The *L. plantarum* GUANKE suspensions were inoculated with 1% (*v*/*v*) inoculum in new MRS medium. The optical density (OD_600_) of the medium was detected at different time points within 24 h, and the growth curve of *L. plantarum* GUANKE was plotted according to the OD_600_ value. The *L. plantarum* GUANKE suspensions (1%) were inoculated in pHs of 3, 5, 7, and 9 and medium containing 0.1%, 0.2%, 0.3%, and 0.4% sterile bovine bile salts (Oxoid, UK) and incubated at 37 °C for different times (3 h, 6 h, 9 h, and 12 h). Based on the detected OD_600_ values, the proliferation curves of *L. plantarum* GUANKE in different environments were plotted. At the same time, plate counting was used to determine the viable bacteria count in the suspension of *L. plantarum* GUANKE [25]. After modifying the method described by Chen, the tolerability of *L. plantarum* GUANKE to the artificial gastrointestinal tract was tested [26]. Briefly, the *L. plantarum* GUANKE grown overnight was collected and resuspended in an equal volume of simulated gastric juice (adjust the normal saline to pH 3.0 using HCL, and add 0.3 g of pepsin after sterilization), incubated at 37 °C for 3 h, and then serial dilution was performed to count the live *L. plantarum* GUANKE. Subsequently, the bacterial fluid after incubation with the simulated gastric juice for 3 h was collected, resuspended in the same volume of simulated intestinal fluid (use NaOH to adjust the normal saline to pH 7.0, and add 0.1 g of trypsin and 0.9 g of bile salt after sterilization), and incubated at 37 °C for 3 h before counting the viable bacteria of the plate. The survival rate of *L. plantarum* GUANKE in artificial gastrointestinal fluid was calculated as follows:

Survival rate (%) = (Nt/N0) × 100%

N0 represents the initial number of viable bacteria, and Nt represents the number of viable bacteria after treatment with simulated gastric juice or simulated intestinal fluid.

### 2.5. Hemolytic Activity Assessment

The β-hemolysis assay of *L. plantarum* GUANKE was carried out on BHI (Brain–Heart Infusion, Basingstoke, UK) agar medium containing 5% defibrinated sheep blood. Briefly, *L. plantarum* GUANKE and *Staphylococcus aureus* isolated in the previous stage of the research (as a positive control for β-hemolysis) after overnight culture were inoculated on BHI agar medium containing 5% defibrinated sheep blood in a three-zone line manner, and the hemolysis of *L. plantarum* GUANKE was evaluated after 48 h of incubation at 37 °C.

### 2.6. Antibiotic Sensitivity Analysis

The sensitivity of *L. plantarum* GUANKE to various antibiotics, including vancomycin, gentamicin, tetracycline, erythromycin, clindamycin, ampicillin, and chloramphenicol, was determined based on the European Union Commission recommendations for probiotic safety (EFSA). The minimum inhibitory concentration (MIC) of these antibiotics against *L. plantarum* GUANKE was ascertained using the E-test method. Briefly, a single colony of *L. plantarum* GUANKE was selected and resuspended in sterile saline to achieve a bacterial suspension with a McFarland turbidity of 0.5. This suspension was then evenly spread onto MRS agar medium using a cotton swab. The test strip was placed on the agar surface before it dried completely, and the plates were incubated at 37 °C for 48 h. Following incubation, the MIC values were interpreted according to the provided instructions (Liofilchem, Teramo, Italy). Strains exhibiting MIC values above the EFSA break-points were considered resistant, while those below were deemed sensitive.

### 2.7. Bacterial Adhesion Capacity Test

Caco-2 cells were used to assess the adhesion ability of *L. plantarum* GUANKE. Caco-2 monolayers (1 × 10^5^ cells/well) were co-cultured with *L. plantarum* GUANKE (1 × 10^8^ CFU/mL) in antibiotic-free DMEM for 4 h. Non-adherent bacteria were removed by PBS washing, followed by cell lysis (0.1% Triton X-100) and viable counts on MRS agar.

### 2.8. In Vitro Cytotoxicity Assays

Lactate dehydrogenase (LDH) release assays and inflammatory cytokine assays were used to assess the vitro toxicity of *L. plantarum* GUANKE. LDH release from Caco-2 cells was quantified using the CytoTox 96^®^ assay (Promega, Madison, WI, USA) after 24 h exposure to *L. plantarum* GUANKE (1 × 10^8^ CFU/mL). Inflammatory cytokines (TNF-α, IL-1β, IL-6) were measured via Enzyme-linked immunosorbent assay (ELISA).

### 2.9. Isolation and Culture of Mice Bone Marrow-Derived Macrophage (mBMDMs) Cells

The tibia and femur of C57BL/6 mice (3~4 weeks) were collected in a biosafety cabinet and the bone marrow cavity was repeatedly rinsed using RPMI1640 medium containing 2% fetal bovine serum until the bone marrow cavity became white and clear. After filtering the collected cell suspension with a cell sieve, erythrocyte lysate was used to lyse the erythrocytes in the cell suspension. Cells were cultured in RPMI 1640 medium containing 100 U/mL penicillin, 100 U/mL streptomycin, 20 ng/mL macrophage-stimulating factor (M-CSF), and 10% fetal bovine serum; half-feeds were performed at intervals of one day; and cells were collected on day 6 for re-counting and plating, and follow-up experiments were performed [27].

### 2.10. In Vitro Anti-Inflammatory Capacity Testing

*L. plantarum* GUANKE with MOIs of 10, 20, and 50 and LPS (1 μg/mL) were co-cultured with mBMDMs cells or THP-1 cells for 24 h, and the LDH content in the culture supernatant was evaluated to determine whether *L. plantarum* GUANKE was cytotoxic. After 24 h of co-incubation with *L. plantarum* GUANKE, mBMDM cells and THP-1 cells were washed with PBS solution to remove bacteria. Subsequently, the cells were co-cultured with LPS solution at a concentration of 1 μg/mL for 4 h, and then centrifuged to collect the culture supernatant and detect the content of inflammatory factors in the supernatant.

### 2.11. Inflammatory Cytokine Assay

ELISA was used to detect the content of inflammatory factors (TNF-α, IL-1β, and IL-6) in the cell supernatant according to the instructions (Thermo Fisher Scientific, Waltham, MA, USA).

### 2.12. Animals

Both animal experiments and animal procedures were approved by the Laboratory Animal Welfare and Ethics Committee of the National Institute for Communication Disease Control and Prevention, Chinese Center for Disease Prevention and Control. Fifteen female C57BL/6J mice (Specific pathogen-free) were purchased from Vital River Laboratory Animal Technology Co., Ltd. and housed at the Laboratory Animal Center of the Chinese Center for Disease Control and Prevention (Certificate No.: SYXK (Beijing) 2017-0021). Of these, five C57BL/6J mice were used to extract mouse bone marrow cells. Ten SPF grade C57BL/6J mice (females) were randomly divided into Control group and GUANKE group, with 5 mice in each group. Mice in the Control group were given 200 μL of sterile PBS solution per day, and mice in the GUANKE group were gavaged with 200 μL of PBS solution containing 2 × 10^9^ CFU *L. plantarum* GUANKE per day. Colon tissue from both groups of mice was collected after one week of continuous gavage.

### 2.13. RNA Sequence

RNA sequencing was performed to examine the effect of *L. plantarum* GUANKE on the colon of mice. As with previous assays [28], transcriptomic analysis of mouse colon samples was performed and analyzed by the BGISEQ-500 system of the Beijing Genomics Institute (BGI). RNA was extracted from mouse colon tissue via the Trizol method (Invitrogen, Carlsbad, CA, USA), and its quality and quantity were assessed using a Fragment Analyzer. Only RNA that met the specified quality criteria was utilized for data library construction. Following mRNA enrichment and targeted amplification, a single-stranded circular DNA library was created and sequenced on the BGISEQ-500 system. Raw data underwent quality control measures to eliminate low-quality sequences, and the remaining sequences were then mapped to the reference genome using HISAT 2 software. Gene Set Enrichment Analysis (GSEA) was carried out on the Dr. Tom analysis platform (https://biosys.bgi.com/, accessed on 10 February 2025). The RNA sequencing data have been deposited in the NCBI SRA database under BioProject No. PRJNA1232951.

### 2.14. Statistical Analysis

Data are expressed as a mean ± SEM. The two-tailed unpaired Student’s *t*-test was used for comparison between the two groups (GraphPad prism v.8.0), and *p* < 0.05 is considered statistically significant (*p* ≤ 0.05, *, *p* ≤ 0.01, **, *p* ≤ 0.001, ***, *p* ≤ 0.0001, ****, ns = not significant).

## 3. Results and Discussion

### 3.1. Genomic Characterization of L. plantarum GUANKE

**Figure 1:**
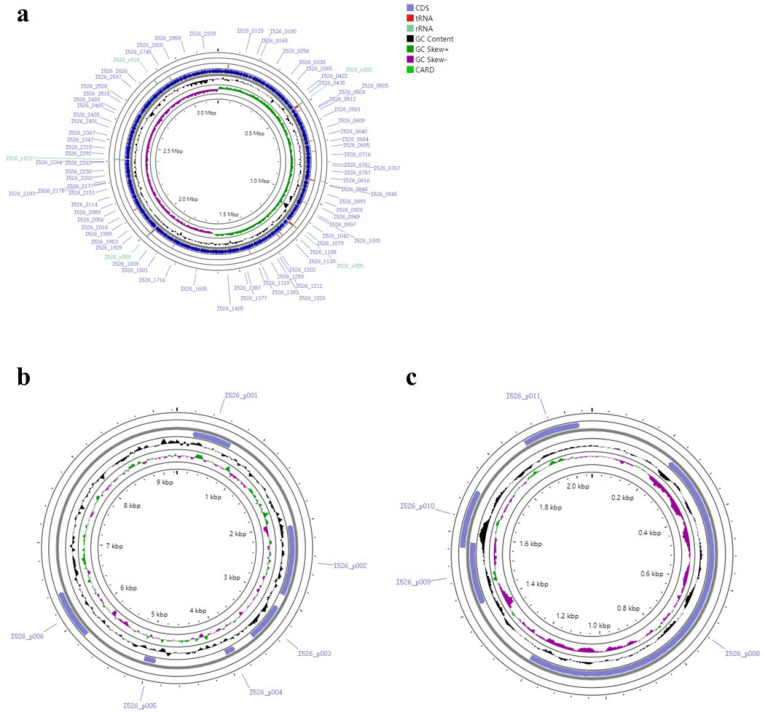
Loop map of the genome of *L. plantarum* GUANKE. (**a**) The genome of the *L. plantarum* GUANKE chromosome and its distribution characteristics. (**b**) Genome overview and distribution characteristics of plasmid (No. CP004407). (**c**) Genome overview and distribution characteristics of plasmid (No. CP004408).

**Figure 2:**
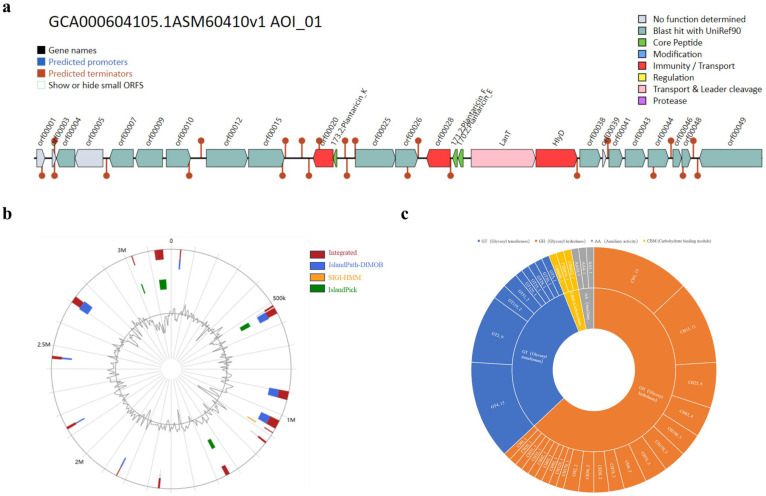
General characteristics of *L. plantarum* GUANKE. (**a**) The bacteriocin gene of *L. plantarum* GUANKE. (**b**) The gene island sequence of *L. plantarum* GUANKE. The red color represents the gene island predicted by the three methods, the blue represents the gene islands predicted by the IslandPath-DIMOB method, yellow represents the gene islands predicted by the SIGI-HMM method, and green represents the gene islands predicted using the IslandPick method. (**c**) Carbenzyme synthesis gene of *L. plantarum* GUANKE.

The *L. plantarum* GUANKE genome consisted of one chromosome (No. CP004406) and two plasmids (No. CP004407 and No. CP004408), with lengths of 3198796 bp, 9253 bp, and 2062 bp, respectively, and the average GC content was 44.7%, 37.4%, and 38.2%, respectively. Figure 1 shows the gene distribution characteristics of the *L. plantarum* GUANKE chromosome and plasmid. The *L. plantarum* GUANKE chromosome contains 2955 CDSs, 66 tRNAs, and 5 rRNAs; the plasmid (No. CP004407) contains six CDSs and the plasmid (No. CP004408) contains four CDSs. The virulence gene prediction of *L. plantarum* GUANKE using VFDB showed that *L. plantarum* GUANKE had no virulence factor. CARD database analysis showed (Table 1) that the *L. plantarum* GUANKE genome may contain vanY and vanH genes related to glycopeptide antibiotic resistance. The CRISPRcasFinder online software was not predicted to have the CRISPR locus and the corresponding Cas gene in *L. plantarum* GUANKE. There are three prophage regions in the *L. plantarum* GUANKE genome, with a length of 18.1 kb~42.7 kb (Table 2). Genes encoding bacteriocins (Plantaricin K, Plantaricin F, and Plantaricin E) were detected in the *L. plantarum* GUANKE genome (Figure 2a). A total of 17 gene islands were predicted in *L. plantarum* GUANKE, and no virulence factor and virulence factor homologs, drug resistance genes and drug resistance gene homologs, and pathogen-related genes were annotated in the predicted gene islands (Figure 2b).

### 3.2. Genomic Function in L. plantarum GUANKE

**Figure 3:**
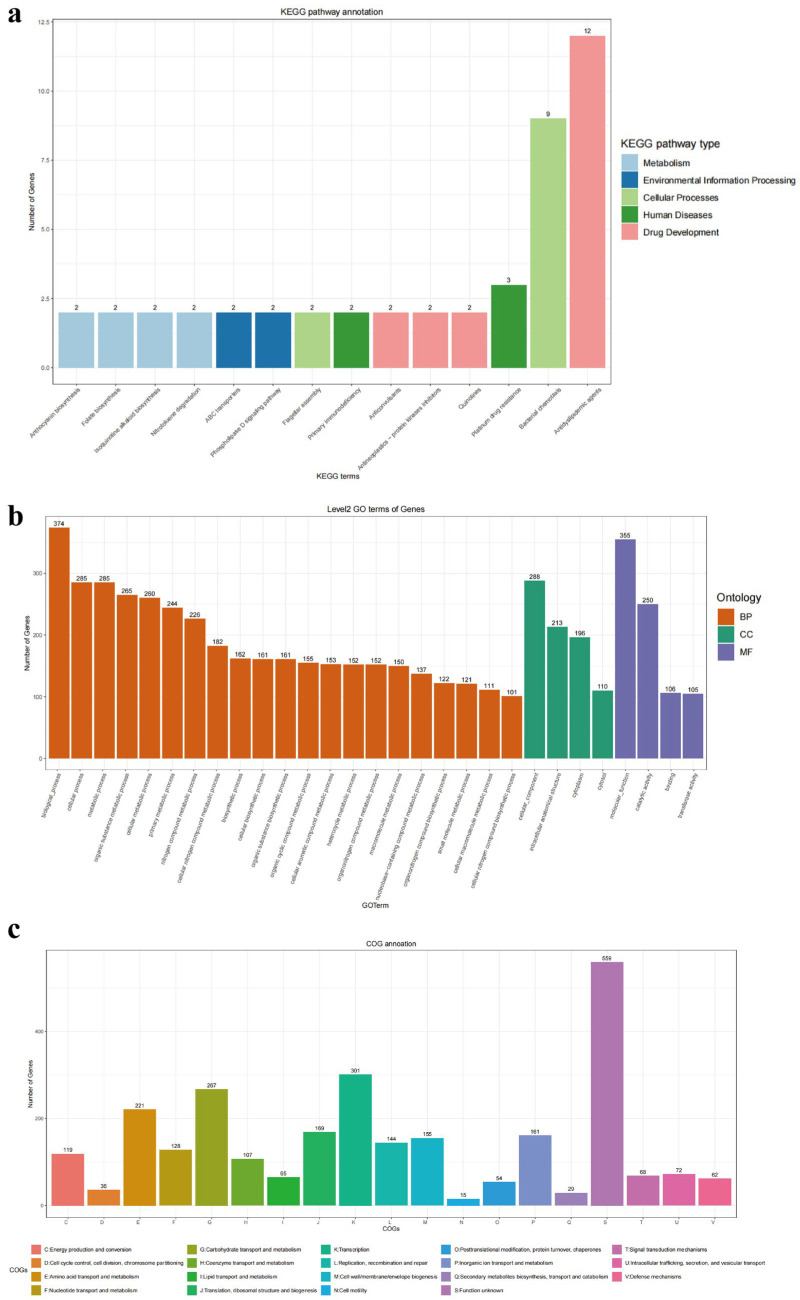
Genome function annotation of *L. plantarum* GUANKE. The eggNOG 5.0 database was used to annotate KEGG (**a**), GO (**b**), and COG (**c**) functions of *L. plantarum* GUANKE’s genes.

As shown in Figure 3, a total of 46 genes in the *L. plantarum* GUANKE genome were annotated by the KEGG database (Figure 3a), of which 18, 5, 11, 4, and 8 genes were related to drug development, human disease, cellular processes, environmental information processing, and metabolism, respectively. A total of 5582 genes in the *L. plantarum* GUANKE genome were annotated by the GO database (Figure 3b), including 3959 genes related to biological processes, 807 genes related to cellular components, and 816 genes related to molecular functions. A total of 2732 genes in the *L. plantarum* GUANKE genome were annotated with COG (Figure 3c), and in addition to the genes with unknown function, the genes involved in transcription, carbohydrate transport and metabolism, and amino acid transport and metabolism were the most numerous, with 301, 267, and 221 genes, respectively. CAZyme analysis of *L. plantarum* GUANKE using the dbCAN server showed (Figure 2c) that *L. plantarum* GUANKE had 100 carbohydrate enzyme activities, including 63 glycoside hydrolases (GHs), 31 glycosyltransferases (GT), and 3 coactive enzymes (AA). The highest number of CAZymes was associated with GH, with 63 genes divided into 21 distinct families. GH has the ability to hydrolyze complex carbohydrates and is considered a key enzyme involved in carbohydrate metabolism. In addition, the analysis showed the presence of three Carbohydrate binding modules (CBMs) in *L. plantarum* GUANKE. Through the analysis of GutSMASH database, it was found that the *L. plantarum* GUANKE genome contained two different classes of metabolism-related gene clusters: one was the E-MGC metabolic cluster and the other was the SCFA metabolic cluster (Table 3).

### 3.3. General Characteristics of L. plantarum GUANKE

**Figure 4:**
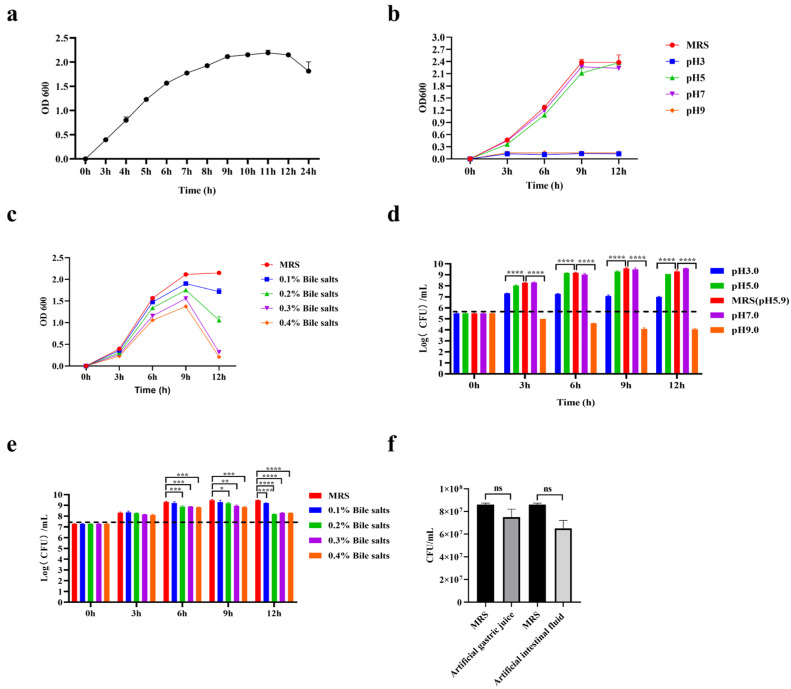
Acid and bile salt tolerance of *L. plantarum* GUANKE. The growth curves of *L. plantarum* GUANKE in normal MRS medium (**a**), MRS medium at different pHs (**b**), and MRS medium with different bile salt concentrations (**c**). The number of viable bacteria in MRS medium of different pHs (**d**) and MRS medium of different bile salt concentrations (**e**). The activity in artificial gastric juice and the activity in intestinal fluid of *L. plantarum* GUANKE (**f**). Data are expressed as a mean ± SEM, statistical analysis was performed by two-tailed Student’s *t*-test (*p* ≤ 0.05, *, *p* ≤ 0.01, **, *p* ≤ 0.001, ***, *p* ≤ 0.0001, ****, ns = not significant).

The results of the growth curve show that *L. plantarum* GUANKE entered the logarithmic growth phase after 3 h of culture and the decline phase after 24 h (Figure 4a). *L. plantarum* GUANKE thrived in MRS medium with a pH range of 5 to 7, proliferated at a relatively slow rate in MRS medium with a pH of 3, and was inhibited in MRS medium with a pH of 9 (Figure 4b,d). After 12 h of incubation, the number of surviving *L. plantarum* GUANKE in a medium with a pH of 9 was only about 3% of the initial quantity. The culture of *L. plantarum* GUANKE in different concentrations of bile salts showed that bile salts had no significant effect on the early proliferation of *L. plantarum* GUANKE (0–9 h), but significantly inhibited the proliferation of *L. plantarum* GUANKE in the later stages of culture (Figure 4c,e). The viability of *L. plantarum* GUANKE in artificial gastrointestinal fluid is shown in Figure 4f. The survival rates of *L. plantarum* GUANKE in artificial gastric juice and artificial intestinal fluid were as high as (87.2 ± 0.03)% and (75.6 ± 0.06)%, respectively.

### 3.4. Hemolysis Analysis, Antibiotic Susceptibility Analysis, and Adhesion Capacity of L. plantarum GUANKE

**Figure 5:**
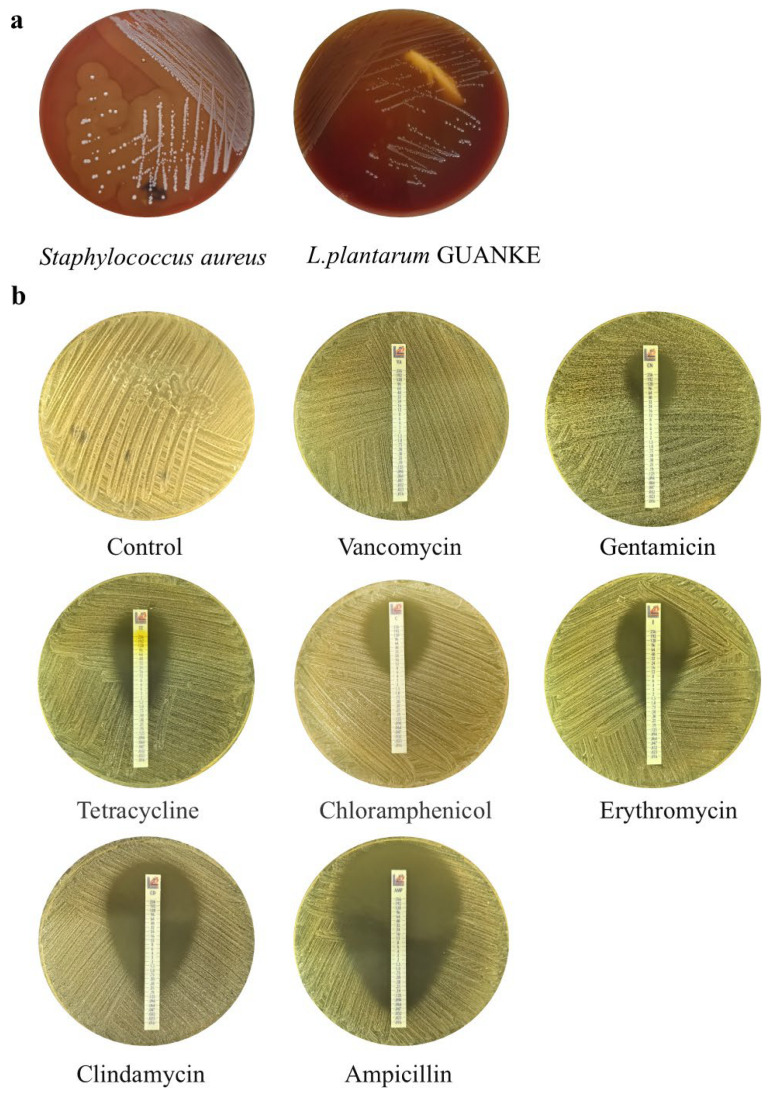
Hemolysis and antibiotic sensitivity of *L. plantarum* GUANKE. (**a**) Hemolytic analysis of *L. plantarum* GUANKE. (**b**) Antibiotic sensitivity analysis of *L. plantarum* GUANKE.

As shown in Figure 5a, *L. plantarum* GUANKE grew well on BHI agar medium containing 5% defibrous sheep blood and did not exhibit significant hemolytic ability, while Staphylococcus aureus grew rapidly on blood medium and formed wide, well-defined, and completely transparent β hemolytic rings around the colonies.

The results of antibiotic susceptibility testing showed that (Figure 5b) *L. plantarum* GUANKE was sensitive to ampicillin, clindamycin, erythromycin, tetracycline, gentamicin, and chloramphenicol, and resistant to vancomycin. Among them, the Minimum Inhibitory Concentration (MIC) value of ampicillin for *L. plantarum* GUANKE was 0.032 μg/mL, the MIC value of clindamycin was 0.38 μg/mL, the MIC value of erythromycin was 1.0 μg/mL, the MIC value of tetracycline was 1.5 μg/mL, the MIC value of gentamicin was 16 μg/mL, the MIC value of chloramphenicol was 6 μg/mL, and the MIC value of vancomycin was greater than 256 μg/mL.

The results of colony counting showed that the average number of colonies adhered to Caco-2 cells was 4.3 × 10^6^ CFU, and the adhesion rate was (4.3 ± 0.007)%.

### 3.5. Cytotoxicity of L. plantarum GUANKE

**Figure 6:**
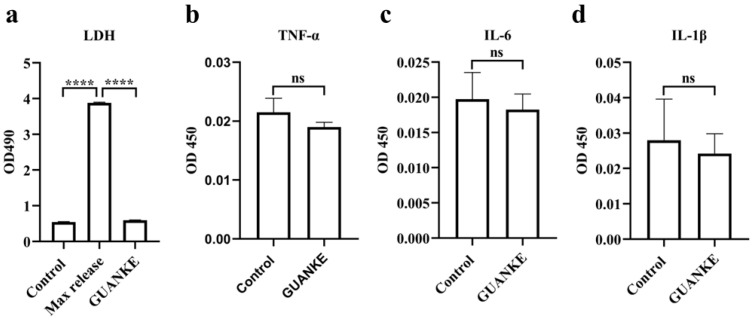
The cytotoxicity of *L. plantarum* GUANKE. After co-incubation with Caco-2 cells for 24 h, the contents of LDH (**a**), TNF-α (**b**), IL-1β (**c**), and IL-6 (**d**) in the cell culture supernatant were detected using a CytoTox 96^®^ assay and ELISA, respectively. Data are expressed as a mean ± SEM, statistical analysis was performed by two-tailed Student’s *t*-test (*p* ≤ 0.0001, ****, ns = not significant).

LDH analysis and the results of ELISA showed that Caco-2 cells did not secrete LDH, TNF-α, IL-1β, and IL-6 significantly after 24 h of co-incubation with *L. plantarum* GUANKE (Figure 6).

### 3.6. In Vitro Anti-Inflammatory Ability of L. plantarum GUANKE

**Figure 7:**
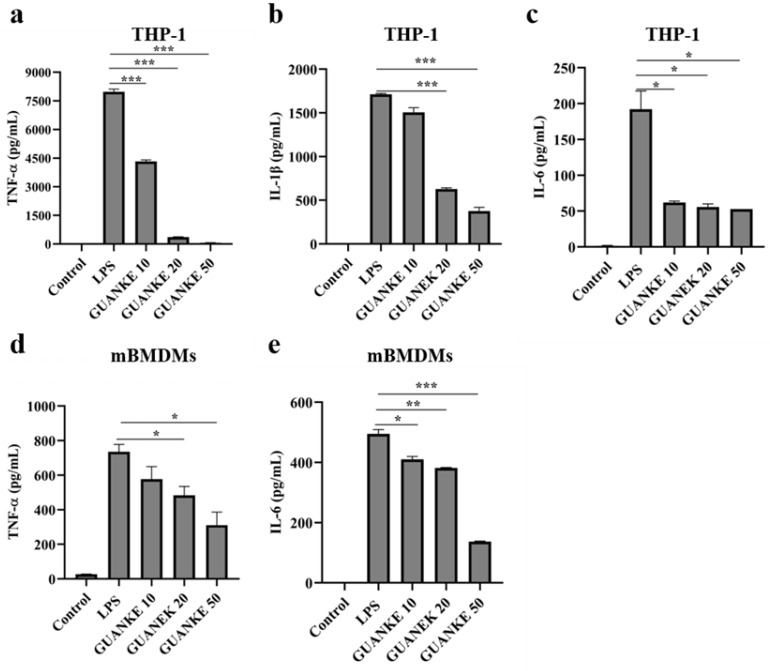
*L. plantarum* GUANKE inhibits secretion of inflammatory cytokines by LPS-induced macrophages. ELISA was used to detect the secretion of TNF-α (**a**), IL-1β (**b**), and IL-6 (**c**) in the supernatant of THP-1 cells and the secretion of TNF-α (**d**) and IL-6 (**e**) in the supernatant of mBMDMs cells after LPS stimulation. Data are expressed as a mean ± SEM, statistical analysis was performed by two-tailed Student’s *t*-test (*p* ≤ 0.05, *, *p* ≤ 0.01, **, *p* ≤ 0.001, ***).

As illustrated in Figure 7, LPS stimulation significantly enhanced TNF-α, IL-1β, and IL-6 secretion in THP-1 cells compared to the control group. Notably, 24 h *L. plantarum* GUANKE pretreatment attenuated LPS-induced cytokine secretion (TNF-α, IL-1β, and IL-6). Among them, the decreases in TNF-α and IL-1β secretion gradually increased with the increase of *L. plantarum* GUANKE concentration, while there was no significant difference in the effect of different concentrations of *L. plantarum* GUANKE on IL-6 secretion. In parallel experiments using mBMDMs cells, LPS challenge significantly induced TNF-α and IL-6 secretion by mBMDMs cells, but failed to induce detectable IL-1β secretion. Compared with the LPS group, *L. plantarum* GUANKE pretreatment with mBMDMs cells for 24 h significantly inhibited LPS-mediated cytokine production in mBMDMs cells.

### 3.7. L. plantarum GUANKE Enhances Apical Junction Pathway and Interferon Response Pathway in Mouse Colon Tissue

**Figure 8:**
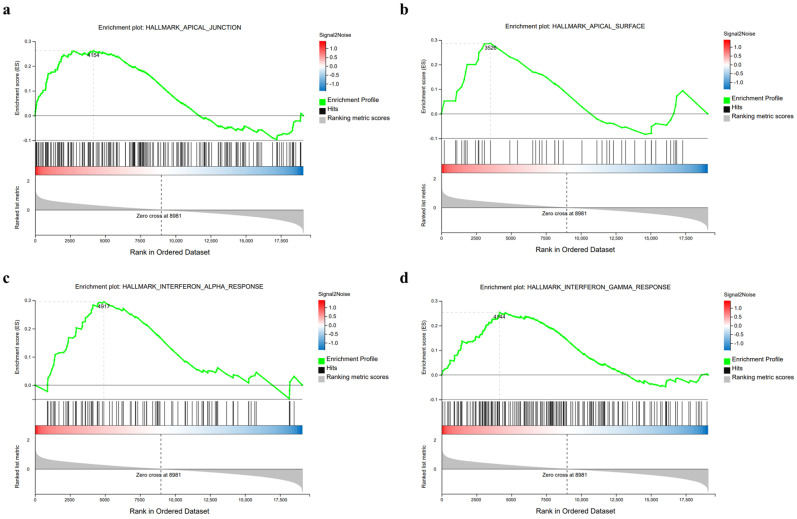
*L. plantarum* GUANKE enhances the apical junction pathway and the interferon response pathway in mouse colon tissue. GSEA analysis revealed that oral administration of *L. plantarum* GUANKE could enhance the apical junction pathway (**a**), apical surface pathway (**b**), interferon-alpha response pathway (**c**) and interferon-gamma response pathway (**d**) in colon tissue.

GSEA analysis of transcriptomic data of mouse colon tissue (Figure 8) showed that the apical junction pathway, apical surface pathway, interferon-alpha response pathway and interferon-gamma response pathway in mouse colon tissue were significantly up-regulated after oral administration of *L. plantarum* GUANKE.

## 4. Discussion

Genome analysis and evaluation of probiotic characteristics are important means to evaluate whether probiotics are suitable for development and application, and the analysis results show that *L. plantarum* GUANKE has good safety and potential probiotic properties.

The genomic analysis of probiotics has mainly included the prediction analysis of virulence factors, antibiotic resistance genes, CRISPR-Cas system, prophages, bacteriocins, gene islands, carbohydrate enzyme synthesis genes, and metabolism-related gene clusters. Virulence factors refer to the molecular or structural components expressed or secreted by pathogens that help pathogens cause host damage during infection, mainly including adhesion factors, invasion factors, toxins, and immune escape factors [29]. The results of this study showed that the annotation of the genome sequence of *L. plantarum* GUANKE using the VFDB database did not reveal any known virulence factors in *L. plantarum* GUANKE. Antimicrobial resistance is one of the major threats hindering the treatment of infectious diseases in humans and animals. Antimicrobial resistance can be acquired in bacteria through genetic mutation and/or horizontal gene transfer [30]. Previous studies have shown that some probiotics carry mobile antimicrobial resistance genes, which may be transferred to other bacteria through horizontal genes after probiotic intervention in the host [31]. In this study, the CARD database was used to predict the genome sequence of *L. plantarum* GUANKE, which showed that the *L. plantarum* GUANKE genome may contain vanY and vanH genes associated with glycopeptide antibiotic resistance (<40%). Antibiotic susceptibility profiling revealed that *L. plantarum* GUANKE exhibited resistance to vancomycin while maintaining susceptibility to ampicillin, clindamycin, erythromycin, tetracycline, and gentamicin. This resistance pattern aligns with established characteristics of *L. plantarum*, which develops intrinsic vancomycin resistance through biosynthesis of peptidoglycan precursors terminating in D-alanyl-D-lactate rather than the conventional D-alanyl-D-alanine substrate. This evolutionary adaptation prevents target binding by vancomycin, a glycopeptide antibiotic that inhibits cell wall synthesis by binding to the D-Ala-D-Ala terminus of peptidoglycan subunits. Notably, this chromosomally encoded resistance mechanism is generally considered not to relate to transmissible antibiotic resistance genes, thereby presenting minimal public health concerns regarding horizontal gene transfer [32,33]. Of particular scientific interest, Song et al. demonstrated that an engineered *L. plantarum* strain producing peptidoglycan with D-alanyl-D-alanine termini triggered significantly elevated secretion of the pro-inflammatory cytokines TNF-α, IL-6, and IL-1β compared to the wild-type strain, suggesting that the natural D-alanyl-D-lactate configuration in lactobacilli may serve as an evolutionary adaptation to mitigate host inflammatory responses [34]. The CRISPR-Cas system, which is primarily composed of CRISPR sequences and Cas genes, is an acquired immune system that is widely present in bacteria and archaea and is used to resist the invasion of exogenous genetic elements such as bacteriophages, plasmids, and viruses. Among probiotic systems, the CRISPR-Cas system is thought to help produce safer and more stable strains, which can enhance the resistance of probiotics to bacteriophages and prevent the spread of plasmids carrying antibiotic resistance markers [35,36]. The CRISPR-Cas system is present in about 63% of lactic acid bacteria, e.g., *L. rhamnosus* LGG contains the CRISPR-Cas9 system [37,38]. Prophages refer to the DNA genome integrated by mild bacteriophages into lysogenic bacterial chromosomes and involved in several life processes of bacteria [39]. On the one hand, it can provide a survival advantage for bacteria in an unfavorable environment, and on the other hand, under specific circumstances, the virulence genes carried by prophages may increase the virulence of the host bacteria and transform non-virulent strains into pathogenic strains [40]. Studies have shown the presence of multiple prophages in a variety of probiotics. Gene islands are relatively independent regions of DNA in the genome of prokaryotes with specific structures and functions, often obtained through horizontal gene transfer. According to the functions of carrying genes, they can be divided into resistance gene islands, pathogenic gene islands, metabolic gene islands, adaptive gene islands, and symbiotic gene islands. The analysis of gene island virulence factors and drug resistance genes of probiotics is an important step to evaluate the safety of probiotics [41,42]. The CRISPR-Cas system, prophage, and gene island prediction analysis of *L. plantarum* GUANKE showed that *L. plantarum* GUANKE did not have a CRISPR locus and corresponding Cas genes, but had three prophage regions and 17 gene islands. Virulence factors and virulence factor homologs, drug resistance genes and drug resistance gene homologs, and pathogen-related genes were not annotated in the predicted gene islands. In addition, hemolytic analysis and cytotoxicity results of bacteria showed that *L. plantarum* GUANKE was not hemolytic and cytotoxic. Together, these results indicate that *L. plantarum* GUANKE has a high safety profile. Bacteriocin is an antimicrobial peptide synthesized by bacterial ribosomes with antimicrobial activity, which can inhibit and kill the same species or related bacteria, and is a potential antibiotic alternative. In addition to inhibiting the growth of intestinal pathogenic bacteria, the bacteriocins produced by probiotics also play a role in inhibiting tumors [43,44,45]. Like other *Lactobacillus plantarum* species, genes encoding the bacteriocins Plantaricin K, Plantaricin F, and Plantaricin E were present in the *L. plantarum* GUANKE genome, suggesting that *L. plantarum* GUANKE may also have the functions of antagonizing pathogen colonization, inhibiting inflammation, and maintaining epithelial barrier integrity [43,44]. Further functional annotation, carbohydrate enzyme synthesis genes, and metabolic-related gene cluster prediction analysis of *L. plantarum* GUANKE using the genome showed that *L. plantarum* GUANKE contained 100 kinds of carbohydrate enzyme activities and two different classes of metabolism-related gene clusters, E-MGC and SCFA. These results suggest that *L. plantarum* GUANKE has good metabolic activity and may play an important role in host health by metabolizing host carbohydrates, participating in pyruvate metabolism and nitrate reduction processes

Tolerance to strong acids in gastric juice and high concentrations of bile salts in the gut is an important prerequisite for probiotics to survive and exert beneficial effects in the host. In our experimental results, although *L. plantarum* GUANKE did not proliferate efficiently in MRS medium at pH 3 or pH 9, it was highly tolerant to bile salts and could proliferate in higher concentrations of bile salt solutions. Artificial gastrointestinal fluid tolerance experiments have once again shown that *L. plantarum* GUANKE has a good tolerance to adverse environments. Although the effect of probiotics does not depend on the long-term colonization of probiotics in the host, good adhesion performance is an important condition for probiotics to exert an ecological competitive advantage. Through in vitro adhesion testing, it was found that the adhesion of *L. plantarum* GUANKE to Caco-2 cells was not significantly better than that of other probiotics [4,46]; the capacity of colonization of *L. plantarum* GUANKE in the gut should be evaluated in vivo in a future study.

Inflammation serves as a crucial protective mechanism in the body, mobilized in response to infection, injury, or stress. However, when inflammation persists or becomes excessive, it can disrupt the delicate balance of tissue homeostasis and evolve into a central pathogenic process in a myriad of diseases. Emerging evidence from human experiments, animal studies, and cell research demonstrates that specific probiotic strains can regulate the inflammatory response. For example, probiotic complexes (*L. casei* Zhang, *L. plantarum* P-8, and *Bifidobacterium animalis* subsp. *lactate* V9) as adjuvant therapy for irritable bowel syndrome significantly reduced serum levels of inflammatory cytokines (IL-6 and TNF-α) in patients [47]. Prophylactic supplementation with acetic acid-producing *Bifidobacterium longum* 5^1A^ significantly attenuated OVA-induced allergic airway inflammation in A/J mice [48]. Administration of *L. paracasei* DG can enhance the antiviral immune response and prevent the inflammatory response elicited by Severe Acute Respiratory Syndrome Coronavirus-2 infection in vitro [49]. The in vitro anti-inflammatory results of this study showed that *L. plantarum* GUANKE could effectively inhibit production of inflammatory cytokines by LPS-induced macrophages. This suggests that *L. plantarum* GUANKE may play an important regulatory role in other inflammatory diseases, like other probiotics.

Furthermore, in order to further evaluate the immune regulation ability of *L. plantarum* GUANKE on colon tissue, transcriptomic analysis was performed on the colon tissue of normal mice following oral administration of *L. plantarum* GUANKE, and the results showed that *L. plantarum* GUANKE was involved in the regulation of colon tissue, including the apical junction pathway, apical surface pathway, interferon-alpha response pathway, and interferon-gamma response pathway. The apical junction complex is a cell–cell adhesion system that is present in the upper part of the outer membrane of epithelial cells, integrated by tight junctions and adherens junctions. This complex is essential for initiating and stabilizing cell–cell adhesion, regulating paracellular transport of ions and molecules, and maintaining cell polarity [50,51]. Multiple strains of probiotics have been demonstrated to exert disease-combating effects by enhancing tight junctions and adherens junctions. For instance, *L. fermentum* KBL374 and KBL375 can ameliorate sodium dextran sulfate-induced colitis by elevating the level of tight junction-related proteins and reducing leukocyte infiltration [52]. *L. rhamnosus* GG safeguards the intestinal mucosa of rats from Pepsin-Trypsin-Digested Gliadin-induced damage by counteracting the decrease of tight junction proteins and adherens junction proteins [53]. Similar to other probiotics, *L. plantarum* GUANKE has the potential to enhance the host’s disease-resistance ability by regulating the apical junction pathway and the apical surface pathway. The interferon system is a pivotal element of the innate immune response, garnering significant attention due to its critical function in the host’s defense against viral infections [54]. Research has revealed that certain probiotic strains possess the ability to bolster the host’s resilience against viruses by modulating the interferon response. A representative example is *L. paracasei* MI29, which confers protection against influenza virus infection via potentiation of type-I interferon-dependent immune responses [55]. Therefore, the ability of *L. plantarum* GUANKE to regulate interferon response in mouse colon tissue suggests that the intervention of *L. plantarum* GUANKE might assist in strengthening the host’s capability to resist viral infections.

## 5. Conclusions

To sum up, in addition to good safety performance and potential probiotic characteristics, *L. plantarum* GUANKE also has the ability to inhibit the inflammatory response, enhance the apical junction pathway and the interferon response pathway, and is a candidate probiotic with development and application potential.

## Figures and Tables

**Table 1:** Potential antibiotic resistance genes in the genome of *L. plantarum* GUANKE.

Gene	Identity	Mode Type	Drug Class	Mechanism	Gene Family
vanY	27.35%	protein homolog model	glycopeptide antibiotic	antibiotic target alteration	glycopeptide resistance gene cluster
vanH	35.02%	protein homolog model	glycopeptide antibiotic	antibiotic target alteration	glycopeptide resistance gene cluster

**Table 2:** Prophage sequence in the genome of *L. plantarum* GUANKE.

Region	Length	Completeness	Total Proteins	Start	End	GC%
1	18.1 kb	questionable	23	37,804	55,910	42.44%
2	38.9 kb	Intact	52	513,739	552,681	41.47%
3	42.7 kb	Intact	53	1,021,684	1,064,390	41.50%

**Table 3:** Analysis of metabolism-related gene clusters in *L. plantarum* GUANKE.

Type	Class	From	To	Core Biosynthetic Gene	Similarity
Nitrate reductase	E-MGC	1,339,351	1,365,822	ctg1_1263, ctg1_1264, ctg1_1265, ctg1_1266	40%
Pyruvate to acetate-formate	SCFA	2,855,852	2,878,968	ctg1_2687, ctg1_2688	100%

## Data Availability

The RNA-sequencing data contained in this study is available in the SRA (Sequence Read Archive) under accession numbers PRJNA1232951 on the NCBI.

## Abbreviations

The following abbreviations are used in this manuscript:

CRISPR	Clustered regularly interspaced short palindromic repeats
Cas	CRISPR-associated
LPS	Lipopolysaccharide
CAZymes	Carbohydrate-active enzymes
FAO	Food and Agriculture Organization of the United Nations (FAO)
WHO	World Health Organization
WGS	Whole-genome sequencing (WGS)
KEGG	Kyoto Encyclopedia of Genes and Genomes
CARD	Comprehensive Antibiotic Resistance Database
PHASTER	Phage Search Tool-Enhanced Release
FBS	Fetal bovine serum
PMA	Phorbol 12-myristate 13-acetate
OD_600_	Optical density 600
MIC	Minimum inhibitory concentration
EFSA	European Union Commission recommendations for probiotic safety
LDH	Lactate dehydrogenase
ELISA	Enzyme-linked immunosorbent assay
M-CSF	Macrophage-stimulating factor
GSEA	Gene Set Enrichment Analysis
